# Increase in tuberculosis among children and young adolescents, European Union/European Economic Area, 2015 to 2023

**DOI:** 10.2807/1560-7917.ES.2025.30.11.2500172

**Published:** 2025-03-20

**Authors:** Veronica Cristea, Csaba Ködmön, Joana Gomes Dias, Senia Rosales-Klintz, the paediatric TB expert group, The paediatric TB expert group, Ágnes Bakos, Anders Koch, Daniela Maria Cirillo, Jean-Paul Guthmann, Jiří Wallenfels, Maria Korzeniewska-Kosela, Mary O’Meara, Monica Sane Schepisi, Nicoleta Cioran, Piret Viiklepp, Raquel Duarte, Sarah Jackson, Stefan Kröger, Teresa Domaszewska, Troels Lillebaek, Walter Haas, Zaida Herrador

**Affiliations:** 1 European Centre for Disease Prevention and Control (ECDC), Stockholm, Sweden; 2 The members of the paediatric TB expert group are listed under Collaborators

**Keywords:** tuberculosis, paediatric tuberculosis, pulmonary tuberculosis, intrathoracic tuberculosis, RR/MDR-TB, pre-XDR-TB

## Abstract

As tuberculosis (TB) in children is an indicator of ongoing transmission, we analysed surveillance data to understand the increase in notified TB cases among individuals aged < 15 years in the European Union/European Economic Area countries between 2015 and 2023. Several factors may have contributed to this increase, such as improved diagnosis and reporting, migration and the COVID-19 pandemic. The observed increasing trend, albeit low in absolute numbers, emphasises the importance of early case finding and timely prevention.

In 2023, 1,689 children and young adolescents (i.e. aged < 15 years) were diagnosed with tuberculosis (TB) (hereafter referred to as paediatric TB) in the European Union/European Economic Area (EU/EEA) countries. Although representing a small percentage of all notified TB cases in 2023, the notification rate in this age group increased from 2.0 per 100,000 population in 2022 to 2.5 per 100,000 population in 2023 [1]. Since paediatric TB is a key indicator of ongoing recent transmission, we analysed the trends and characteristics of paediatric TB cases notified in the EU/EEA between 2015 and 2023 to understand the recent increases.

## A snapshot of paediatric tuberculosis

On 1 October 2024, we extracted case-based surveillance data on notified TB cases 2015–2023 from the European Surveillance System (TESSy) of the European Centre for Disease Prevention and Control (ECDC). We included all confirmed, probable and possible TB cases notified by 28 countries, using pre-established case definitions [2] and analysed the data by age (< 1, 1–4, 5–9 and 10–14 years). Data from Liechtenstein and Latvia were not available in TESSy for the whole study period, thus they were excluded from this analysis.

**Figure 1:**
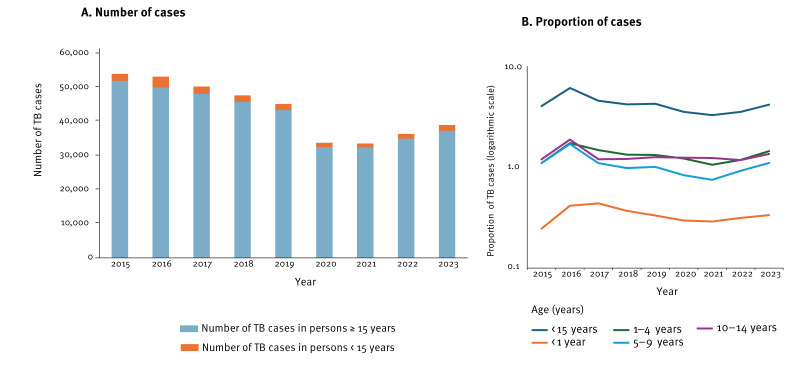
Number of notified tuberculosis cases and proportion of paediatric tuberculosis cases (n = 16,414) among all tuberculosis cases (n = 393,104), European Union/European Economic Area countries, 2015–2023

In total, 16,414 paediatric TB cases were notified. Overall, the paediatric TB cases constituted a small proportion of all TB cases (n = 393,104) notified in the EU/EEA (ranging from 3.4% in 2021 to 6.4% in 2016), with an average of 1,946 cases per year (ranging from 1,142 in 2021 to 3,126 in 2016). We observed a fluctuating trend, with a substantial 37% decrease between 2019 (n = 1,810) to 2021 (n = 1,142) and a gradual increase between 2021 and 2023 (n = 1,689) (Figure 1).

TB: tuberculosis.

Paediatric tuberculosis was defined as tuberculosis in persons aged < 15 years.

The highest proportion of paediatric TB cases was observed among children aged 1–4 years (n = 5,394; 32.9%) (Figure 1), and pulmonary TB was the predominant clinical manifestation across all age groups (n = 9,179; 55.9%). The proportion of laboratory-confirmed paediatric cases (n = 4,710) of the total number of reported cases was 28.7% (from 2016: 668 of 3,126 cases; 21.4% to 2021: 387 of 1,142 cases; 33.9%) while individuals without a laboratory confirmation accounted for 11,704 (71.3%) cases (from 2016: 2,458 cases; 78.6% to 2021: 755 cases; 66.1%).

Among the paediatric cases with laboratory confirmation (according to the EU case definition [2]), drug-resistant paediatric TB was rare: 107 (2.3%) isolates were rifampicin-resistant (RR) or multidrug-resistant (MDR)-TB (MDR-TB defined as resistance to isoniazid and rifampicin), 16 (0.3%) were pre-extensively drug-resistant (XDR)-TB (i.e. MDR-TB with resistance to any fluoroquinolone) and one isolate (0.02%) was XDR-TB (i.e. pre-XDR-TB with additional resistance to either bedaquiline or linezolid) [3]. Italy was excluded from this analysis since disaggregated MDR-TB per specific age groups studied was not available.

## Clinical and geographical patterns across age groups

**Figure 2:**
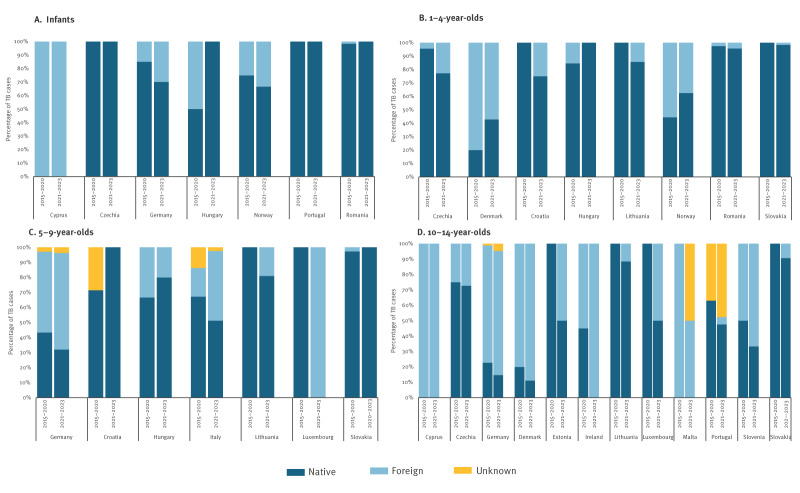
Paediatric tuberculosis, by age and origin, European Union/European Economic Area countries, 2015–2023 (n = 16,414)^a^

To investigate the increase in paediatric TB after 2021, we characterised each age group by comparing the proportion of paediatric TB cases among all TB cases for two periods: the mean proportion of notifications 2015–2020 vs yearly data for 2021–2023. In the further analyses, we included countries reporting a proportion of ≥ 5% increase in the overall paediatric TB notifications in at least one age group (Table). We stratified paediatric TB cases by country of origin as native (born in or citizen of the reporting country) or foreign (born in or citizen of a country different from the reporting country) (Figure 2).

**Table:** Paediatric tuberculosis cases in countries with an observed increase of ≥ 5% 2015–2020 (n = 2,732) and 2021–2023 (n = 1,260), by age, notification rates and increase, European Union/European Economic Area countries, 2015–2023

Reporting country	Total number notified		Mean 2015–2020		2021		2022		2023		Changes 2015–2020 and 2021–2023	
	2015–2020	2021–2023	NR	P (%)	NR	P (%)	NR	P (%)	NR	P (%)	NR^a^	P (%)^a^
Infants (aged < 1 year)												
Cyprus	2	1	3.59	0.56	0.00	0.00	0.00	0.00	9.66	1.23	0.28	21.74
Czechia	5	6	0.52	0.17	1.86	0.56	0.00	0.00	3.92	0.87	0.52	50.43
Germany	114	47	2.47	0.36	1.56	0.31	1.64	0.32	3.04	0.49	0.05	8.00
Hungary	2	2	0.38	0.05	0.00	0.00	0.00	0.00	2.24	0.41	0.56	67.61
Norway	4	3	1.15	0.09	0.00	0.00	1.77	0.16	3.85	0.28	0.35	33.49
Portugal	39	19	7.58	0.12	8.27	0.19	6.29	0.12	8.35	0.16	0.02	6.69
Romania	193	84	16.08	1.68	12.16	1.56	15.54	1.72	18.63	2.11	0.04	5.87
1–4-year-olds												
Croatia	2	4	0.22	0.12	1.42	1.14	0.00	0.00	1.41	0.75	0.45	58.92
Czechia	23	22	0.86	0.79	1.11	1.40	0.90	1.05	2.82	2.83	0.27	37.58
Denmark	20	7	1.39	1.14	1.21	1.38	0.40	0.43	1.19	1.55	-0.03	8.05
Hungary	13	5	0.59	0.33	0.80	0.90	0.00	0.00	0.54	0.41	-0.02	5.70
Lithuania	38	14	5.43	0.50	0.00	0.46	0.00	0.27	0.00	1.24	0.10	25.35
Norway	18	8	1.24	1.33	1.31	0.00	1.31	2.89	1.28	1.96	0.00	10.26
Romania	834	306	17.92	1.13	5.16	0.87	6.04	0.89	4.64	1.63	-0.05	9.67
Slovakia	157	65	11.34	10.47	8.44	9.49	10.27	10.32	19.92	16.29	0.02	11.69
5–9-year-olds												
Croatia	7	1	0.57	0.28	0.00	0.00	0.00	0.00	0.57	0.38	0.00	7.65
Germany	277	134	1.28	0.87	0.71	0.69	1.16	1.10	1.28	1.38	0.04	12.44
Hungary	3	5	0.11	0.08	0.22	0.30	0.66	0.68	0.11	0.20	0.15	25.93
Italy	226	92	1.36	1.07	0.85	0.89	0.83	0.86	1.36	1.69	0.08	12.17
Lithuania	67	21	7.89	0.92	2.10	0.46	2.76	0.54	7.89	1.94	0.03	20.59
Luxembourg	1	2	0.52	0.92	0.00	0.00	0.00	0.00	0.52	4.35	0.61	65.85
Slovakia	74	54	4.29	5.08	5.17	10.95	4.44	8.39	4.29	11.76	-0.15	23.35
10–14-year-olds												
Cyprus	1	2	0.38	0.67	0.00	0.00	0.00	0.00	4.10	2.47	0.61	67.40
Czechia	4	11	0.13	0.45	0.52	0.84	0.86	1.31	0.50	0.65	0.31	45.07
Denmark	30	9	1.49	0.16	0.59	0.92	0.30	0.43	1.82	3.11	0.04	15.50
Estonia	3	2	0.74	0.60	0.00	0.00	0.00	0.00	2.51	2.02	0.28	60.03
Germany	356	211	1.61	0.16	1.58	1.50	1.58	1.45	2.45	2.08	0.09	16.49
Ireland	20	6	1.04	0.18	0.28	0.49	0.00	0.00	1.32	2.24	0.05	18.42
Lithuania	81	26	10.19	0.10	494	1.08	5.53	1.08	7.33	1.52	-0.06	9.68
Luxembourg	1	2	0.53	0.43	0.00	0.00	2.93	2.08	2.84	2.17	0.40	42.93
Malta	5	1	3.85	0.14	0.00	0.00	0.00	0.00	4.31	1.47	0.02	13.66
Portugal	73	42	2.33	0.12	2.40	0.78	2.81	0.89	3.23	1.02	0.07	12.31
Slovakia	37	43	2.32	0.39	3.88	8.03	4.56	8.39	6.56	8.60	0.23	39.44
Slovenia	2	3	0.36	0.43	0.91	1.25	0.89	1.35	0.87	1.14	0.19	43.30

NR: notification rate per 100,000 population; P: proportion of paediatric tuberculosis cases of all tuberculosis cases reported to the European Surveillance System (TESSy).

^a^ Change in mean of 2015–2020 compared with 2021–2023.

Paediatric tuberculosis was defined as tuberculosis in persons aged < 15 years.

TB: tuberculosis.

^a^ Countries reporting ≥ 5% increase in the mean proportion of notifications of paediatric tuberculosis 2015–2020 versus 2021–2023 in at least one age group.

Paediatric tuberculosis was defined as tuberculosis in persons aged < 15 years. Infants were defined as children aged < 1 year, natives were born in or citizen of the reporting country) and foreign were born in or citizen of a country different from the reporting country.

Of the 17 countries with ≥ 5% increase in the proportion of paediatric TB notifications in at least one age group, five reported increases in one age group (Estonia, Ireland, Italy, Malta and Slovenia), seven reported increases in two age groups (Croatia, Cyprus, Denmark, Luxembourg, Portugal, Norway and Romania) and five reported increases in three age groups (Czechia, Germany, Hungary, Lithuania and Slovakia) (Table). No country reported increases in all four age groups.

Tuberculosis notifications among infants (i.e. children aged < 1 year) and young children were predominantly among native children, whereas young adolescents (i.e. aged 10–14 years) had a diverse origin (Figure 2). In five countries (Czechia, Croatia, Lithuania, Slovakia and Romania), increases were almost exclusively among native children (Figure 2).

Clinical manifestations varied across age groups and countries. Between 2021 and 2023, Czechia reported increases in intrathoracic TB among infants (3 of 6 cases) and young children (14 of 22 cases). In contrast, Romania consistently reported higher numbers of intrathoracic TB in young children (2015–2020: n = 453; 54.3% and 2021–2023: n = 173; 56.5%) compared with the other countries.

Drug-resistant paediatric TB was rare in these 17 countries. Of 3,992 cases, 37 (0.9%) isolates were RR/MDR-TB, seven (0.2%) were pre-XDR-TB and none were XDR-TB.

## Predictive trend analysis on the paediatric cases

**Figure 3:**
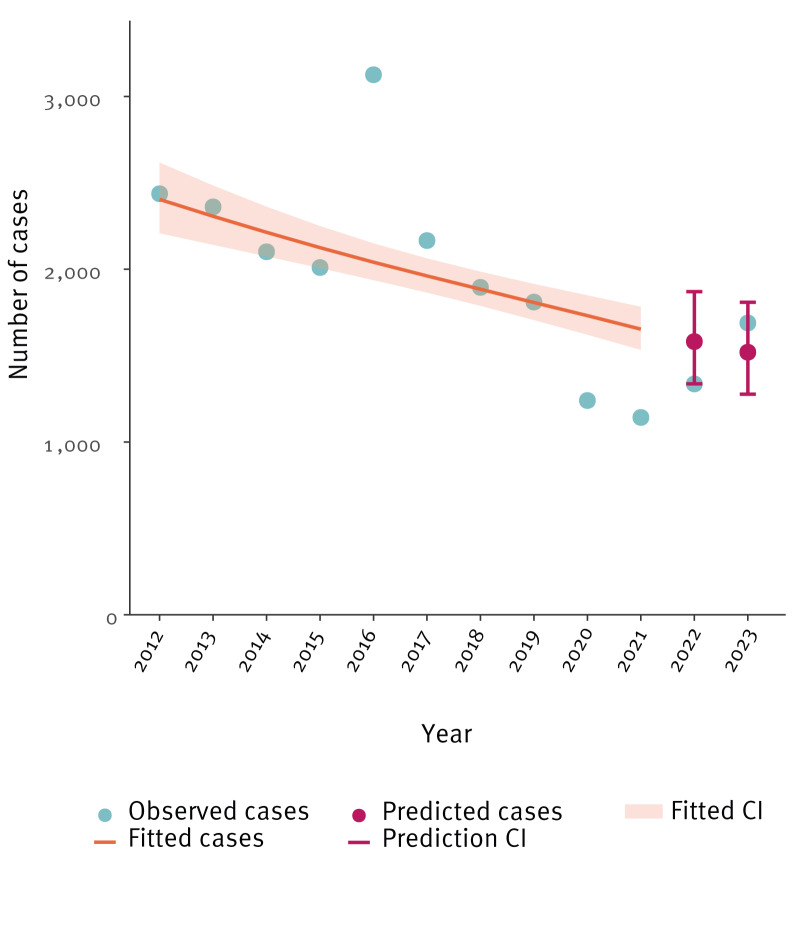
Trend of paediatric tuberculosis, European Union/European Economic Area, 2012–2023 (n = 23,313)

To further assess the increases observed 2012–2023, we conducted a predictive analysis by fitting a negative binomial regression model using packages MASS_7.3-61, readr_2.1.5 and tidyverse_2.0.0 in R statistical programme (https://www.r-project.org/) version 4.4.2. To minimise the influence of outliers, we expanded the analysis period (from 2012 onwards) and excluded data from 2016, 2020 and 2021. The 2016 data were excluded due to an unusual increase in notified cases (France reported > 30% of all paediatric cases; n = 1,153). Data from 2020 and 2021 were excluded due to the COVID-19 pandemic disruptions. The predicted trend assuming that the years 2016 and 2020–2021 had no long-term effect on TB incidence, showed a decrease. However, the observed values in 2022 (n = 1,336) were at the lower upper-bound of the confidence interval (CI) (predicted value = 1,581 cases, 95% CI: 1,337–1,871), while in 2023, the observed cases (n = 1,689) were above the predicted number but within the expected CI (predicted value = 1,520 cases, 95% CI: 1,277–1,809) (Figure 3).

CI: confidence interval.

Paediatric tuberculosis was defined as tuberculosis in persons aged < 15 years.

## Discussion

We sought to understand the increase in paediatric TB observed in 2022 and 2023 in the EU/EEA countries, but our analysis of TB surveillance data could not single out a specific explanation for the increase. The observed increases were small and seen in low-incidence countries (< 10 cases per 100,000 population per year) [4]. As such, even a minor change in the number of notified cases can result in an observed proportional increase.

Several factors probably influenced this increase, e.g. improved diagnosis and reporting of paediatric TB, social risk factors among native children, and changes in population movements [5]. Additionally, the impact of the COVID-19 pandemic on healthcare services [6,7] might have also played a role. Our predictive trend analysis might suggest higher notification rates due to the delayed diagnosis in adult cases 2020–2021. If the upward trend continues, increased TB transmission is a likely cause.

The United States (US), the United Kingdom (UK) and Canada also reported significant increases in TB notifications among children and adolescents in 2022 and 2023. In the US, paediatric TB mostly affects children born in the country, often with parents coming from high-incidence regions [8,9]. In the UK, the TB notification rate was higher in non-UK born children, who often had concomitant social risk factors (e.g. asylum seeker status and history of homelessness) [10]. In Canada, on the other hand, the incidence of paediatric TB was higher in indigenous populations [11]. Our data lacked information on the parents’ country of origin, social determinants and contact tracing. Access to this type of data as part of national routine surveillance, would enhance the knowledge on the contribution of existing transmission and social pattern in the EU/EEA. Previous studies have demonstrated the influence of such factors in the epidemiology of paediatric TB in the EU/EEA [12-15].

Other limitations of our study are the lack of information on the children’s Bacillus Calmette-Guérin (BCG) vaccination status and travel history to or from high-incidence countries. Additionally, the impact of people displacement due to Russia’s war in Ukraine was not evaluated. Finally, over- or underestimation of the number of cases may have occurred due to variation in case definitions used by the reporting EU/EEA countries and the use of the revised 2021 World Health Organization (WHO) definition for pre-XDR and XDR-TB [3].

## Conclusion

This analysis of paediatric TB strengthens the understanding of the burden of paediatric TB in the EU/EEA and suggests the continued impact of COVID-19 pandemic management on early diagnosis on the overall trends. Despite the increasing proportion of paediatric TB cases reported in some EU/EEA countries 2022–2023, the numbers of notified paediatric cases remain relatively low. Strengthened surveillance, prompt contact tracing and preventive measures are needed to limit the potential ongoing TB transmission.
